# Sleep duration and quality of Brazilian nursing staff who work in shifts

**DOI:** 10.1590/0034-7167-2023-0167

**Published:** 2024-06-14

**Authors:** Rosângela Marion da Silva, Regina Célia Gollner Zeitoune, Flávia Camef Dorneles Lenz, Carolina Renz Pretto, Katerine Moraes dos Santos, Tânia Solange Bosi de Souza Magnago, Alexa Pupiara Flores Coelho Centenaro

**Affiliations:** I Universidade Federal de Santa Maria. Santa Maria, Rio Grande do Sul, Brazil; II Universidade Federal do Rio de Janeiro. Rio de Janeiro, Rio de Janeiro, Brazil; III Universidade Federal de Santa Maria. Palmeira das Missões, Rio Grande do Sul, Brazil

**Keywords:** Nursing, Team, Shift Work Schedule, Hospitals, Public, Sleep, Signs and Symptoms, Equipe de Enfermagem, Jornada de Trabalho em Turnos, Hospitais Públicos, Sono, Sinais e Sintomas, Grupo de Enfermeira, Horário de Trabajo por Turnos, Hospitales Públicos, Sueño, Signos y Síntomas

## Abstract

**Objective:**

to analyze sleep duration and sleep quality in nursing professionals who work in shifts.

**Method:**

this is a cross-sectional, analytical research, carried out between September 2017 and April 2018, at a public hospital in southern Brazil, with the nursing team. A socio-occupational and health symptoms questionnaire, the Epworth Sleepiness Scale, and the Pittsburgh Sleep Quality Index were used. Data are presented as descriptive and inferential statistics, bivariate analysis, and binary logistic regression.

**Results:**

participants were 308 nursing professionals with a predominance of long-term sleep, absence of drowsiness, and poor sleep quality. Short-term sleep (<6h) was associated with day shift and poor sleep quality. Sleep quality was associated with presence excessive daytime sleepiness and work day shift.

**Conclusion:**

work shift, insomnia and headache were the main factors related short-term sleep for nursing professionals. The results may justify the development of intervention research for workers’ health.

## RESUMO

**Objetivo:**

analisar a duração e qualidade do sono em profissionais de enfermagem que trabalham em turnos.

**Método:**

pesquisa transversal, analítica, realizada entre setembro de 2017 e abril de 2018, em hospital público do Sul do Brasil, com equipe de enfermagem. Foram utilizados questionário sócio-ocupacional e de sintomas de saúde, Escala de Sonolência de Epworth e Índice de Qualidade do Sono de Pittsburgh. Os dados são apresentados como estatística descritiva e inferencial, análise bivariada e regressão logística binária.

**Resultados:**

participaram 308 profissionais de enfermagem com predomínio de sono prolongado, ausência de sonolência e má qualidade do sono. Sono de curta duração (<6h) foi associado a turno diurno e má qualidade do sono. Qualidade do sono esteve associada à presença de sonolência diurna excessiva e trabalho diurno.

**Conclusão:**

turno de trabalho, insônia e cefaleia foram os principais fatores relacionados ao sono de curta duração para profissionais de enfermagem. Os resultados podem justificar o desenvolvimento de pesquisas de intervenção para a saúde do trabalhador.

## RESUMEN

**Objetivo:**

analizar la duración y calidad del sueño en profesionales de enfermería que trabajan por turnos.

**Método:**

investigación analítica, transversal, realizada entre septiembre de 2017 y abril de 2018, en un hospital público del sur de Brasil, con el equipo de enfermería. Se utilizó el cuestionario sociolaboral y de síntomas de salud, la Escala de Somnolencia de Epworth y el Índice de Calidad del Sueño de Pittsburgh. Los datos se presentan como estadística descriptiva e inferencial, análisis bivariado y regresión logística binaria.

**Resultados:**

participaron 308 profesionales de enfermería, con predominio de sueño prolongado, ausencia de somnolencia y mala calidad del sueño. La duración corta del sueño (<6 h) se asoció con turnos de día y mala calidad del sueño. La calidad del sueño se asoció con la presencia de somnolencia diurna excesiva y con el trabajo diurno.

**Conclusión:**

los turnos de trabajo, el insomnio y la cefalea fueron los principales factores relacionados con la falta de sueño de los profesionales de enfermería. Los resultados pueden justificar el desarrollo de investigaciones de intervención para la salud de los trabajadores.

## INTRODUCTION

The nursing team’s mental and physical health is related to the commitment to patient work, care, and well-being^(1)^, and can be adversely affected by work in the day and night shifts in hospital settings. The work carried out in hospital institutions contrasts with increased levels of stress when compared to non-hospital service^(2)^, and suggests worker exposure to long working hours, which can have repercussions on their health^(3)^.

In Brazil, nursing work is carried out by nurses, nursing technicians, and nursing assistants, who carry out their activities following the Professional Practice Law and face daily challenges inherent to the work process. The requirement of working day and night shifts suggests sleep impairment and has the potential for the development of negative manifestations in nursing professionals’ health due to non-synchronization with circadian rhythms. Although these rhythms are strongly driven by the light/dark cycle, humans have individualized sleep time preferences based on genetics and external influences^(4)^. Atypical hours of sleep and wakefulness expose workers to excessive sleepiness and/or insomnia^(5)^, poor sleep quality, and the occurrence of physical and psychological harm^(6)^.

Sleep is an essential component of people’s health and well-being. Sleep restriction can negatively affect the nursing team’s health and patient safety^(7,8,9,10)^. Studies have identified that short sleep duration was associated with night shift^(7)^, age greater than or equal to 50 years, having more than one child^(8)^, being single^(7, 8)^, having memory problems^(9)^, having symptoms of depression^(11)^, poor quality of care and patient safety^(10)^, chronic discomfort in the neck and shoulders^(12)^, low mood and high levels of stress in nurses^(13)^.

The studies presented so far contribute to understanding the relationship between sleep and some associated factors in nursing professionals in some countries. But what are sleep duration and sleep quality in Brazilian nursing professionals? The nature of nursing work cannot be changed, nor is exposure to risks of illness^(14)^. However, the need to fill this research gap to generate knowledge that promotes evidence-based interventions is emerging to contribute to workers’ health, with repercussions on quality of care.

## OBJECTIVE

To analyze sleep duration and sleep quality in nursing professionals who work in shifts.

## METHODS

### Ethical aspects

The ethical and legal principles postulated in Resolution 466/12 were followed. The study was approved by the Research Ethics Committee. All participants provided written Informed Consent Form, signed the document and returned to the researchers.

### Design study, period and place

The study followed the recommendations to improve observational research description quality and EQUATOR Network guidelines, using the STrengthening the Report of OBservational studies in Epidemiology (STROBE).

This is a cross-sectional, analytical study, carried out at a university tertiary care hospital in southern Brazil.

This hospital was selected considering that it is a reference for the region covered by the population, with more than 1,000,000 inhabitants distributed in 42 municipalities, and the researchers’ affiliation.

### Sample; inclusion and exclusion criteria

Nurses (higher level), nursing technicians, and nursing assistants (technical level) participated in this study. According to information from the institution, at the time of data collection, the population comprised 960 nursing professionals. The minimum representative sample size of the population was 277 nursing professionals, considering a 95% confidence level and a 5% sampling error. The sample was randomly stratified by professional category, considering the finite population.

After calculating the minimum sample, the proportion of each category in the total population was verified (35% of nurses, 52% of nursing technicians, and 13% of nursing assistants), defining the minimum representative sample of 97 nurses, 144 nursing technicians, and 36 nursing assistants.

Professionals were invited individually to their workplaces. We included nursing professionals who worked in day and night shift care, and who were not in management positions, and we excluded those who were on vacation or leave of any kind during the period of data collection. The end of collection occurred when the minimum sample was reached. If there was a participation agreement, the data collection instruments were sent, and a return date was scheduled. When the questionnaires were returned, verification of the complete completion of items ensured that there was no missing data in the variables of interest. A collection instrument not returned after the fifth collection attempt was considered a loss. Based on the identified population, 350 questionnaires were distributed, with a response rate of 88% (n=308). There were 10 refusals and 32 losses.

Seven members of the research group, undergraduate students, scientific initiation scholarship holders, and graduate students, all in nursing, who were trained in person for this stage of the research, and who received a manual to minimize doubts, helped in data collection. Meetings for the delivery of completed questionnaires and clarification of doubts occurred weekly.

### Study protocol

Data collection was carried out from September 2017 to April 2018 in adult and child hospitalization units, intensive care and adult and child emergency rooms, surgical center, and post-anesthetic recovery.

### Analysis of results, and statistics

A questionnaire was used for socio-occupational characterization and identification of health symptoms. It is a self-report instrument with the variables age, job tenure, position (nurse, nursing technician/assistant), work shift (day and night), medication use (yes and no), physical activity (yes and no), health symptoms: irritability, insomnia, headache, feeling of depression, feeling of low self-esteem (once a week and never).

The Pittsburgh Sleep Quality Index (PSQI-BR) was used, in the translated and validated version for Brazilian Portuguese^(15)^, which has 19 questions and seven components, such as sleep quality, latency to sleep, sleep duration, habitual sleep efficiency, sleep disorders, use of sleep medication, daytime dysfunction. The higher the score, the worse the sleep quality. An overall score of 5 indicates that the person has major difficulties with at least two components or moderate problems with three or more components. We chose to categorize the variable sleep quality into “good” (≤ 5) and “poor” (> 5)^(6)^, and the variable sleep duration into ≥ 6 h (long duration) and < 6 h (short duration)^(7)^ according to previous studies.

We used the Epworth Sleepiness Scale (ESS-BR), validated for the Brazilian Portuguese version^(16)^. It is a self-administered scale, consisting of eight questions that present situations of drowsiness in daily life, and must be answered considering the chance of falling asleep. The sum of items ≤ 10 was considered the absence of excessive daytime sleepiness, and the sum > 10 was the presence of excessive daytime sleepiness^(17)^.

Data were double entered and statistically analyzed using statistical tests of the PSS package (Predictive Analytics Software, SPSS INc., Chicago, USA) version 18.0 for Windows. Categorical variables were assessed using absolute (n) and relative (%) frequencies. Quantitative variables were presented as mean ± standard deviation (SD). For associations between exposure and outcome variables, the chi-square test was used, with 95% confidence levels.

Data collection instruments are widely used in studies with nursing professionals. Reliability was assessed through internal consistency analysis by Cronbach’s alpha coefficient (ESS-BR=0.82; PSQI-BR= 0.72).

Binary logistic regression (using the Enter method) was used to identify the adjusted association between the short sleep duration variable (dependent variable) and other variables. Logistic regression models were run with the variables, which were removed from the models as p-value was greater than 25%. The associations of all models were expressed with Odds Ratios, p-values, and 95% confidence intervals.

Multicollinearity was checked using the variance inflation factor (VIF), and a VIF < 5 (or 10) for each variable was acceptable.

We checked the goodness of fit using the Hosmer-Lemeshow test. For the binary regression, the variables that obtained p<0.25 in bivariate analysis entered the adjusted model 1, and in adjusted model 2, p<0.15 with statistical significance (p<0.05).

## RESULTS

The final sample had 308 nursing professionals, greater than the estimated sample size. Of the participants, 32.5% (n=100) were nurses and 67.5% (n=208) were nursing technicians and assistants; 54.9% (n=169) worked on the day shift and 45.1% (n=139) on the night shift. There were 86.4% (n=266) female; 77.9% (n=240) said they had a partner; and 72.7% (n=224) had children. The mean age was 40.84 years (±9.13), with a minimum of 23 years and a maximum of 69 years. Moreover, 45.5% (n=140) used medication and 49% (n=151) did not practice physical activity.

Poor sleep quality was prevalent (84.7%, n=261), in addition to absence of daytime sleepiness (58.8%, n=181). A predominance of long-term sleep (≥ 6 h) was identified (84.4%, n=260).

When associating sleep duration and excessive daytime sleepiness, no significant association was identified (p=0.481). Regarding the components of the instrument that assesses sleep quality regarding sleep latency, which is the time needed to fall asleep, we identified that workers with long-lasting sleep (≥ 6 h) took up to 30 minutes to fall asleep, while those with short duration sleep (< 6 h) took more than 30 minutes to fall asleep (p=0.010).

The sleep efficiency component was associated with sleep duration, showing a significant association between short sleep and worse sleep efficiency (less than 85%), and between long sleep duration and efficiency of 85% or more (p<0.001).

Table 1 shows the distribution of workers according to sleep duration, sleep quality, excessive daytime sleepiness, socio-occupational variables and health symptoms. Sleep quality was associated with sleep duration and excessive daytime sleepiness (p<0.05).

**Table 1:** Distribution of workers according to sleep duration, sleep quality, excessive daytime sleepiness, socio-occupational variables and health symptoms. Santa Maria, RS, Brazil, 2017-2018 (N=308)

Variables	Sleep duration		Χ^2^ *	Sleep quality		Χ^2^ *
	≥ 6 h	< 6 h		Good	Poor	
Excessive daytime sleepiness						
Absence	155(85.6)	26(14.4)	0.481	34(18.8)	147(81.2)	0.040
Presence	105(82.7)	22(17.3)		13(10.2)	114(89.8)	
Work shift						
Day	133(78.7)	36(21.3)	0.002	19(11.2)	150(88.8)	0.031
Night	127(91.4)	12(8.6)		28(20.1)	111(79.9)	
Medication use						
Yes	110(78.6)	30(21.4)	0.010	18(12.9)	122(87.1)	0.284
No	150(89.3)	18(10.7)		29(17.3)	139(82.7)	
Physical activity						
Yes	142(90.4)	15(9.6)	0.003	27(17.2)	130(82.8)	0.335
No	118(78.1)	33(21.9)		20(13.2)	131(86.8)	
Irritability						
Once a week	186(81.6)	42(18.4)	0.020	29(12.7)	199(87.3)	0.036
Never	74(92.5)	6(7.5)		18(22.5)	62(77.5)	
Insomnia						
Once a week	121(77.1)	36(22.9)	<0.001	9(5.7)	148(94.3)	<0.001
Never	139(92.1)	12(7.9)		38(25.2)	113(74.8)	
Headache						
Once a week	158(79.4)	41(20.6)	0.001	21(10.6)	178(89.4)	0.002
Never	102(93.6)	7(6.4)		26(23.9)	83(76.1)	
Feelings of depression or unhappiness						
Once a week	112(79.4%)	29(20.6)	0.027	10(7.1)	131(92.9)	<0.001
Never	148(88.6)	19(11.4)		37(22.2)	130(77.8)	
Feelings of decreased health						
Once a week	121(79.6)	31(20.4)	0.022	10(6.6)	142(93.4)	<0.001
Never	139(89.1)	17(10.9)		37(23.7)	119(76.3)	

* p < *0.05*

An association was identified between short-term sleep (< 6 h) and poor sleep quality, work day shift, medication use, physical activity, and among the health symptom variables such as irritability, insomnia, headache, feelings of depression or unhap-piness and feelings of decreased health (p < 0.05).

We also found that sleep quality was associated with excessive daytime sleepiness, work shift and the variables of health symptoms such as irritability, insomnia, headache, feelings of depression and feeling of low self-esteem (p < 0.05).

Table 2 shows the unadjusted and adjusted associations between short-duration sleep, sleep quality and variables.

**Table 2:** Adjusted associations between short-duration sleep, sleep quality and variables. Santa Maria, RS, Brazil, 2017-2018 (N=308)

	Unadjusted association OR (CI)	†AdjLBR 1*	‡AdjLBR 2**	‡RBLAjust 3**
Sleep quality				
Poor	10.10(1.35-75.11)	0.46(0.60-36.45)	5.26(0.68-40.69)	
Good	1	1	1	
Work shift				
Day	2.86(1.43-5.75)	2.29(1.10-4.77)	2.34(1.14-4.83)	2.48(1.19-5.02)
Night	1	1		
Irritability				
Once a week	2.78(1.14-6.83)	1.58(0.59-4.20)	-	-
Never	1	1		
Insomnia				
Once a week	3.45(1.72-9.92)	2.36(1.13-4.91)	2.51(1.22-5-19)	2.92(1.43-5.97)
Never	1	1		
Headache				
Once a week	3.78(1.63-8.75)	2.38(0.98-5.76)	2.61(1.09-6.21)	2.83(1.20-6.71)
Never	1	1		
Feelings of depression or unhappiness				
Once a week	2.02(1.08-3.78)	1.34(0.62-2.90)	-	-
Never	1	1		
Feelings of decreased health				
Once a week	2.09(1.10-3.97)	1.07(0.48-2.40)	-	-
Never	1	1	-	-

Note: †AdjLBR 1 (p<0.25): Adjusted logistic binary regression 1: sleep quality + work shift + irritability + insomnia + headache + feelings of depression or unhappiness + feelings of decreased health. *Hosmer and Lemeshow test = 0.674 ‡AdjLBR 2 (p<0.15): Adjusted logistic binary regression 2: sleep quality + work shift + insomnia + headache **Hosmer and Lemeshow test = 0.946†††AdjLBR 3 (p<0.10): Adjusted logistic binary regression 3: work shift + insomnia + headache. **Hosmer and Lemeshow test = 0.881.

Multivariate models showed that day shift were 2.48 twice as likely to have short-duration sleep in nursing professionals. Nursing professionals with insomnia and headache one or more times a week were more likely to have short-duration sleep (OR=2.92, CI= 1.43-5.97; OR=2.83, CI = 1.20-6.71, respectively).

## DISCUSSION

We identified the prevalence of long-term sleep, poor sleep quality, and absence of excessive daytime sleepiness, although a significant amount showed drowsiness.

There was a significant relationship between sleep duration < 6 h and work shift, sleep quality, medication use, physical activity and symptom of irritability, insomnia, headache, feelings of depression or unhappiness and feelings of decreased health. Sleep quality was associated with excessive daytime sleepiness, work shift and the variables of health symptoms irritability, insomnia, headache, feelings of depression and feeling of low self-esteem. Furthermore, there are higher chances for work shift, insomnia and headache health symptoms for those with short-duration sleep.

Regarding socio-occupational variables, the sample consisted of professionals who, for the most part, were young adults, females, nurses, having a partner, and children.

There was a prevalence of professionals who did not practice physical activity, with statistical significance being identified with short-term sleep. Additionally, there was a significant relationship between those who practiced physical activity and long-term sleep. This data converges with the result of a study carried out with nurses in Saudi Arabia, which found that lack of exercise was significantly associated with short sleep duration^(11)^. We suggest physical activity encouraged by hospital managers to promote nursing professionals’ health and improve sleep quality.

Day shift was significantly associated with short-term sleep, and regression analysis showed that work day shift increased the odds of nursing professionals by 2.45 times to have a short-term sleep. This data differs from studies thatidentified that short sleep duration was associated with night shift^(7)^ and research carried out with nurses working in hospitals in Taiwan, which did not identify an association between sleep duration and work shift^(12)^. The work performed during the day shift at the institution studied in this research involves multiple tasks such as referrals for complementary exams, surgical procedures, involvement with visitors, ample movement, and high levels of attention and concentration. This requires nursing professionals to use relaxation strategies that collaborate with their health.

There was a significant relationship between medication use and short sleep. Data from a Norwegian study carried out with nurses identified a strong association between those with less than 7 hours of sleep and use of prescribed and non-prescribed drugs to sleep^(18)^. Despite the importance of this data in this study, we highlighted a research limitation of the non-investigation on the type of medication used by nursing professionals, which would allow other analyses.

As for the variables of health symptoms, we identified that the feeling of depression one or more times a week was significantly related to sleep duration < 6 h. This data is like research that identified greater chances of nurses with a duration of ≤ 5 h of sleep having symptoms of depression when compared to nurses with 6 or 7 hours of sleep per day^(11)^, which may have implications for workers’ health and quality of care. The sector that monitors workers’ health in health institutions should consider the assessment of symptoms self-reported by workers that are recurrent but nonspecific, such as constant sadness, loss of interest in routine activities, low energy, pain without a defined cause, low self-esteem, suggesting compromising workers’ mental health and that they need periodic follow-up.

There was a significant difference between the feeling of low self-esteem, having short sleep duration, and having poor sleep quality. Self-esteem is understood as an individual’s assessment of their value, a determinant of social behavior and results in life (interpersonal relationships, work, and health)^(19)^. Therefore, having a short duration of sleep or having daytime sleepiness suggests workers to negative thoughts and emotional states, with possible consequences on health and repercussions on patient safety.

Irritability was associated with having a short sleep duration and poor sleep quality. Research carried out with Spanish nurses identified higher mean scores in stress and mood management among those who slept 7 hours or more. This suggests that it is essential to make health professionals aware of the importance of sleep quality for well-being and to develop programs that promote healthy sleep habits^(13)^.

We identified that nursing professionals who sleep < 6 h a day had increased chances of having a headache. This data is confirmed by Chinese researchers, who identified headache as the risk factor most strongly associated with sleep problems, while regular diet and exercise were the factors that protected the study population against sleep problems^(8)^.

Furthermore, the data highlighted that having a short duration of sleep reflects a greater chance of having insomnia, showing that sleep restriction harms workers’ health. American research suggests that nurses who sleep less may be more fatigued to work, resulting in impaired performance^(10)^. This data is reinforced by a Norwegian study that shows the relationship between short sleep and worse memory, which can compromise the instructions received, with negative effects on health care^(9)^. From this, it is recommended that immediate supervisors consider the health condition presented by workers and develop collective actions to improve these people’s health to prevent quality of work from being impaired.

When sleep duration was associated with time to fall asleep and sleep efficiency, we identified that those with < 6 h of sleep took more than 30 minutes to fall asleep and had worse sleep efficiency (< 85%), which points to the need to prepare the environment and adopt habits that prepare the body for rest such as reducing exposure to light, adopting regular sleep schedules and paying attention to the type of meals and drinks. Sleep efficiency reflects the calculation between the actual hours of sleep and the time that a person remained in bed to sleep^(20)^, i.e., the amount of time that one remains asleep.

A short sleep pattern may be related to a deterioration in the subjective quality and perceived effectiveness of sleep^(13)^. Additionally, long-term sleep enables recovery and promotes well-being as well as the practice of physical activity. From this, we highlighted the importance of maintaining healthy habits and leisure and rest activities that positively impact these workers’ health conditions^(19, 21)^.

Sleep quality was associated with short sleep duration and presence excessive daytime sleepiness. Sleep quality is defined as individuals’ self-satisfaction with all aspects of the sleep experience. Consequences of poor sleep quality include fatigue, daytime dysfunction, slow responses, increased caffeine/alcohol intake, and irritability^(22)^.

Sleep problems will affect workers’ physical and mental recovery, and people with sleep problems may experience mood changes. Nursing professionals should pay attention to signs and symptoms that may be having an unfavorable impact on health and take measures to mitigate them.

Finally, considering the negative influences of sleep on nursing professionals’ health, which can have repercussions on care, and that day and night shift work is not avoidable, interventions should be implemented to improve these professionals’ quality of sleep and health. We need to consider that workers’ health is influenced by work organization, with shift work being an aspect that can contribute to workers’ illness^(23)^. It is important to raise awareness of workers to self-care, disseminate information about the factors that interfere with sleep and repercussions on health, proposing preventive actions and monitoring the early recognition of signs and symptoms that may be contributing to worsened health.

### Study limitations

Regarding the limitations of this research, this study was based on self-reports on socio-occupational data, health, and sleep symptoms, which can cause reporting bias due to the potential for recall. Furthermore, the cross-sectional design does not allow for establishing causal relationships, and future longitudinal data analyzes will be needed to verify the study findings. It is mentioned that using questionnaires that showed satisfactory internal consistency, with Cronbach’s alpha values greater than 0.70, and which are widely reproduced in research, can contribute to reducing the subjectivity of responses and allow comparison with other studies, which helps to mitigate these limitations.

Finally, considering that the Brazilian population is quite heterogeneous and that there are social, cultural, and economic differences, this study carried out at a single hospital institution can contribute to identifying situations that can also be identified in other regions of the country and the world, allowing comparisons.

### Contributions to nursing, health, and public policies

The results provide additional information on sleep assessment in these professionals and its repercussions on workers’ health and health care. They may also justify the development of intervention research for workers’ health. The practice of physical activity seems to be a beneficial factor for quality of sleep. Therefore, it can be encouraged and included among nursing professionals’ health-promoting activities.

## CONCLUSION

In this study, the analysis of sleep duration and sleep quality of Brazilian nursing professionals showed that sleeping less than 6 hours a day is related to poor sleep quality, day shift, not performing physical activity, and having one or more times a week symptom of irritability, depression and low self-esteem. Sleep quality with presence excessive daytime sleepiness, work day shift and having one or more times a week symptom of irritability, depression and low self-esteem. Work shift, insomnia and headaches increases the chances of short-term sleep.

## Data Availability

https://doi.org/10.48331/scielodata.93EALF
